# A novel hybridization capture method for direct whole genome sequencing of clinical specimens to inform Legionnaires’ disease investigations

**DOI:** 10.1128/jcm.01305-23

**Published:** 2024-03-21

**Authors:** Phillip Weeber, Navjot Singh, Pascal Lapierre, Lisa Mingle, Danielle Wroblewski, Elizabeth J. Nazarian, Wolfgang Haas, Don Weiss, Kimberlee A. Musser

**Affiliations:** 1 Wadsworth Center, New York State Department of Health, Albany, New York, USA; 2 New York City Department of Health and Mental Hygiene, New York, New York, USA; Medical College of Wisconsin, Milwaukee, Wisconsin, USA

**Keywords:** *Legionella*, NGS, direct, WGS, specimens, autopsy, investigation, relatedness

## Abstract

The unprecedented precision and resolution of whole genome sequencing (WGS) can provide definitive identification of infectious agents for epidemiological outbreak tracking. WGS approaches, however, are frequently impeded by low pathogen DNA recovery from available primary specimens or unculturable samples. A cost-effective hybrid capture assay for *Legionella pneumophila* WGS analysis directly on primary specimens was developed. DNA from a diverse range of sputum and autopsy specimens PCR-positive for *L. pneumophila* serogroup 1 (LPSG1) was enriched with this method, and WGS was performed. All tested specimens were determined to be enriched for *Legionella* reads (up to 209,000-fold), significantly improving the discriminatory power to compare relatedness when no clinical isolate was available. We found the WGS data from some enriched specimens to differ by less than five single-nucleotide polymorphisms (SNPs) when compared to the WGS data of a matched culture isolate. This testing and analysis retrospectively provided previously unconfirmed links to environmental sources for clinical specimens of sputum and autopsy lung tissue. The latter provided the additional information needed to identify the source of these culture-negative cases associated with the South Bronx 2015 Legionnaires’ disease (LD) investigation in New York City. This new method provides a proof of concept for future direct clinical specimen hybrid capture enrichment combined with WGS and bioinformatic analysis during outbreak investigations.

**IMPORTANCE:**

Legionnaires’ disease (LD) is a severe and potentially fatal type of pneumonia primarily caused by inhalation of *Legionella*-contaminated aerosols from man-made water or cooling systems. LD remains extremely underdiagnosed as it is an uncommon form of pneumonia and relies on clinicians including it in the differential and requesting specialized testing. Additionally, it is challenging to obtain clinical lower respiratory specimens from cases with LD, and when available, culture requires specialized media and growth conditions, which are not available in all microbiology laboratories. In the current study, a method for *Legionella pneumophila* using hybrid capture by RNA baiting was developed, which allowed us to generate sufficient genome resolution from *L. pneumophila* serogroup 1 PCR-positive clinical specimens. This new approach offers an additional tool for surveillance of future LD outbreaks where isolation of *Legionella* is not possible and may help solve previously unanswered questions from past LD investigations.

## INTRODUCTION

*Legionella* species are intracellular opportunistic bacterial pathogens present in the environment and commonly found in freshwater environments such as lakes and streams. The presence of *Legionella* species becomes a health concern in engineered water systems like cooling towers, air conditioning systems, and other urban water systems. Legionnaires’ disease (LD) is a severe and potentially fatal type of pneumonia primarily caused by inhalation of contaminated aerosols from man-made water or cooling systems. The species *Legionella pneumophila* is responsible for more than 90% of LD cases worldwide (1). LD presents a serious risk for individuals who are over 50 yr old, smokers, those with weakened immune systems or pre-existing respiratory conditions. Most LD patients recover with antibiotic treatment but approximately 10% die from the infection (2).

Since the start of the COVID-19 pandemic in 2019, various coinfections with other pathogens including *L. pneumophila* have been reported in patients with COVID-19 (3, 4). Increased LD risk in office and retail buildings opened after the pandemic lockdown has also been reported (5). LD remains vastly underdiagnosed as it is an uncommon form of pneumonia, and its diagnosis relies on clinicians requesting *Legionella* testing. Furthermore, it is challenging to obtain clinical lower respiratory specimens from cases of legionellosis, and when available, culture requires specialized media and growth conditions, which are not available in all microbiology laboratories. For all of these reasons, it is often difficult to link cases to the source of an outbreak, hindering identification and remediation of outbreak sources. Hence, there are continuous efforts to develop and utilize new tools for *Legionella* genomic-based testing.

*Legionella* testing has rapidly evolved since initial cases of LD were described more than 45 yr ago. Improvements have included refined testing algorithms, culture techniques, real-time PCR, pulsed-field gel electrophoresis (PFGE), and more recently, whole genome sequencing (WGS), a type of next-generation sequencing (NGS) in ongoing investigations of LD (6). WGS has become an essential method to support LD outbreak investigations in recent years due to its high discriminatory power. However, bacterial isolates are not always available.

We recently developed a method to enrich Shiga toxin-producing *Escherichia coli* (STEC) genomic DNA directly from stool specimens by hybrid capture (7). The technique was developed to maintain public health disease surveillance with the increasing use of culture-independent diagnostic testing. In the current study, a similar and improved method for *L. pneumophila* hybrid capture by RNA baiting was developed, which allowed us to generate sufficient genome resolution from *L. pneumophila* serogroup 1 (LPSG1) PCR-positive clinical specimens. Initial testing included clinical sputum specimens with a diverse range of LPSG1 real-time PCR cycle threshold (Ct) values. Our data showed that the enriched baited specimens matched very closely to the WGS of their pure isolate pairs. In 2015, New York City detected an increase in LD cases in the South Bronx (SB) caused by LPSG1. The outbreak occurred in a low-income community with a high prevalence of comorbidities and health conditions (8). This SB outbreak was one of the deadliest in the United States history, with 138 cases identified: 128 of these requiring hospitalizations, and 16 individuals ultimately dying from LD (9, 10). An extensive investigation, involving multiple health agencies, was able to link the outbreak to a particular cooling tower, leading to the subsequent mitigation. A closed genome of *L. pneumophila*, strain F4468, isolated from the cooling tower source of the South Bronx outbreak, resulted from our investigation. This investigation (10) also included the analysis of 289 cooling tower water samples from 183 cooling towers, with 162 (88.5%) cooling towers positive for *Legionella* species DNA. LPSG1 DNA was detected in 52 (28.4%) cooling towers, many closely related by WGS and bioinformatic analysis to the outbreak strain, among these was a strain, ESCH2A, from a cooling tower.

Although many isolates from patients were recovered and linked to this cooling tower, no isolates were recovered from some of the LD deaths, and to this date, these cases could not be linked to this outbreak with WGS analysis. *Legionella* species in autopsy specimens can be refractory to the recovery by culturing, severely limiting the WGS-based analysis. Here, we tested the discriminatory power of *L. pneumophila* hybrid capture on a subset of unresolved LD autopsy specimens from the SB and identified the source of outbreak for some of these cases. This new approach offers an additional tool for surveillance of future LD outbreaks where isolation of *Legionella* is not possible and may help solve previously unanswered questions from past LD investigations.

## MATERIALS AND METHODS

### Sputum and autopsy specimen study groups

We randomly selected 30 real-time PCR-positive sputum specimens in our clinical specimen archive that met the criteria of Ct values ranging from 20 to 38. Additionally, a matched pure isolate was recovered from 27 of these 30 specimens for which additional testing was performed and could be matched to the specimen data.

As part of the 2015 SB outbreak, we received autopsy specimens from 13 cases including fresh and formalin-fixed right and left lung tissue; vitreous fluid and urine were collected in a collaborative effort between the New York State and the New York City. DNA isolated from six archived fresh lung tissue autopsy specimens from suspect cases from the 2015 SB outbreak was examined in this hybrid capture study.

### Specimen DNA extraction

DNA from fresh and formalin-fixed tissue (0.2–0.3 g), vitreous fluid (200 µL), and urine (1 mL, when available) specimens was extracted following a modified Epicentre Masterpure Complete DNA and RNA Purification Kit method (10). Briefly, for 200 µL of fluid sample, volumes of reagents were adjusted to 1.5 µL Proteinase K, 200 µL of 2X T and C lysis buffer, 200 µL MPC protein precipitation reagent, and 667 µL isopropanol (rinsed once). Samples were resuspended in 40 µL 10 mM Tris, and 5 µL of internal control reagents was added at the cell lysis stage. For tissues, we followed our modified procedure as indicated above but started by grinding fresh or formalin-fixed tissue in Feeley Gorman Broth (1.5 mL) starting with 0.3-mg tissue, and the internal control reagent was adjusted to 10 µL.

### Real-time PCR screen

DNA extracts were tested using an updated multiplex real-time PCR assay that detects the *Legionella* genus, *L. pneumophila* serogroups 1–15, and specifically LPSG1 (11, 12). In addition, an internal control reagent is incorporated to assess for inhibition and extraction efficiency. Ct values were determined.

### Culture

Culture of tissue homogenates ground in Feeley Gorman broth was performed for all PCR-positive specimens. Each sample was then plated onto blood agar, buffered charcoal yeast extract agar (containing L-Cysteine hydrochloride and a-Ketoglutarate [BCYEα], BCYEα with polymyxin B, anisomycin, and vancomycin [BCYEα+ PAV]), and BCYEα with bromocresol blue and bromothymol green dyes, glycine, vancomycin, and polymyxin B. In some cases, an acid wash pretreatment (0.2 mol/L HCL-KCL, pH 2.2, for 3 min) or heat pretreatment (50°C for 10 min) was utilized before plating. Ethanol treatment was performed for cultures overgrown with swarming organisms (13).

### WGS of pure isolates

Genomic DNA from isolates recovered from culture was extracted with modifications using the Epicentre MasterPure Complete DNA and RNA Purification Kit (10) as described above. DNA was quantitated using the Qubit dsDNA BR assay system. The Nextera XT DNA Sample Preparation Kit was utilized to prepare sequencing libraries. Sequences were generated using the Illumina MiSeq System.

### Whole genome RNA baits

**Fig 1:**
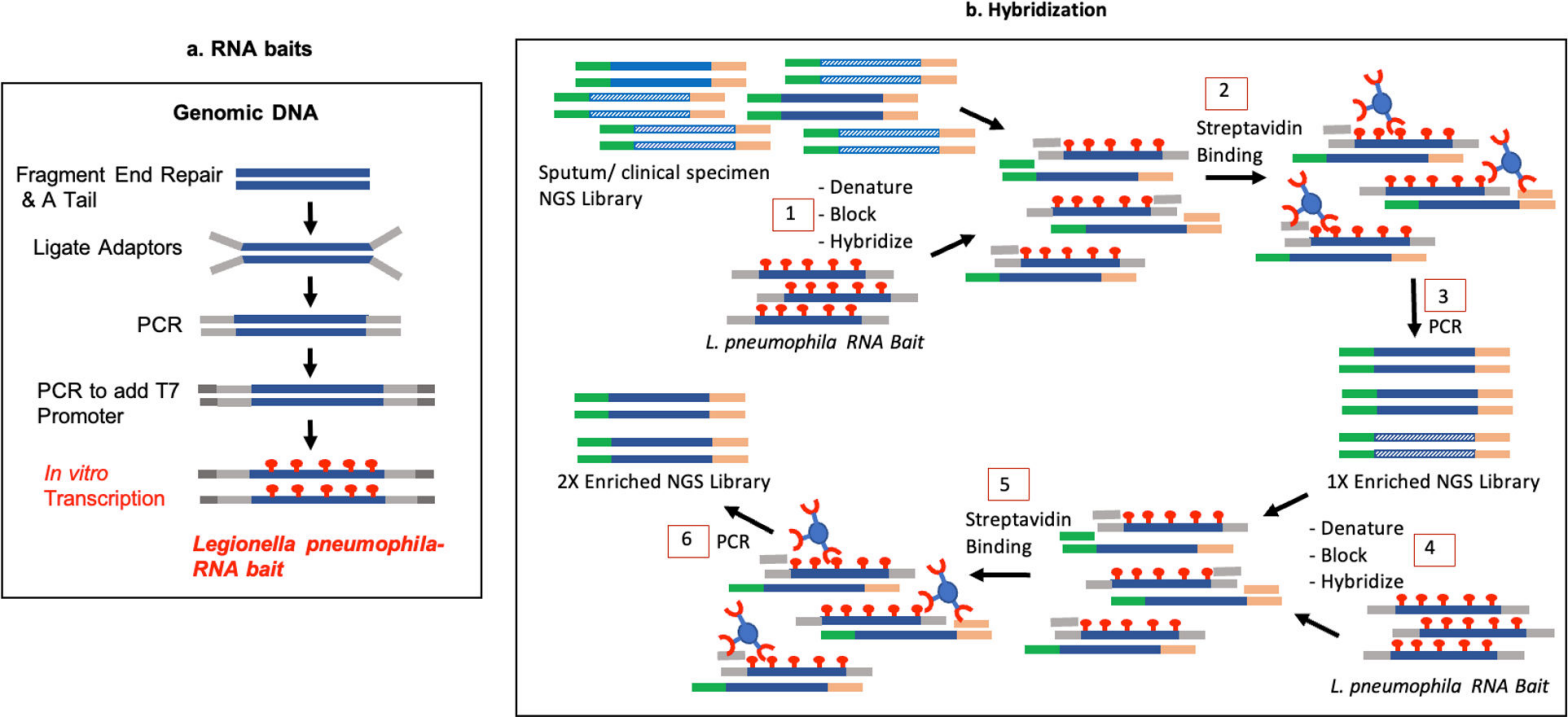
Improved workflow for *L. pneumophila* hybrid capture. (a) The DNA templates for RNA baits were prepared using DNA Library Prep Kit for Illumina using a DS adaptor (gray), and T7 promotor (dark gray) was added using PCR. RNA capture baits (dark blue) were generated by *in vitro* transcription reaction with biotin labeled UTP (red). (b, step 1) NGS library for primary samples (blue bars with green and beige Illumina adaptors) was denatured, blocked with DS adaptor and T7 oligonucleotides, and hybridized to the capture baits. (b, step 2) Hybridized *L. pneumophila* DNA was enriched by binding to streptavidin beads. (b, step 3) Samples were amplified and subjected to a second round of enrichment (2X) (b, steps 4, 5, and 6) prior to WGS.

An improved workflow for enrichment of *Legionella* by hybrid capture was developed (7, 14), and modifications are described in Fig. 1. In a pilot assay, a two-strain RNA bait template pool was generated by mixing equal proportion of *L. pneumophila* Philadelphia and *L. pneumophila* Knoxville genomic DNA. Next, a five-strain RNA bait template pool was prepared by mixing *L. pneumophila* Paris, *L. pneumophila* Toronto-2005, *L. pneumophila* F4468 (isolated from the cooling tower source of the South Bronx outbreak), *L. pneumophila* Dallas, and *L. pneumophila* Philadelphia. RNA baits were prepared from ~70 ng total pooled DNA. The fragmentation, end repair and A tailing was carried out in one reaction using the NEBNext UltraII FS DNA Library Prep Kit for Illumina (New England Biolabs, Ipswich, MA, USA) on a thermocycler with conditions: 37°C for 30 min, 65°C for 30 min. The adaptor ligation was performed with double-stranded (DS) adaptors instead of the Illumina adaptors. The DS adaptors were generated by mixing an equimolar ratio of oligonucleotides 5′-TGTAACATCACAGCATCACCGCCATCAGTCxT-3′ (where “x” is exonuclease I-resistant phosphorothioate linkage) and 5’[PHOS]GACTGATGGCGCACTACG ACACTACAATGT-3′, heating to 95°C for 5 min and cooling to 22°C at a ramp speed of 0.1°C/s, and were cleaned using AmpureXP (Beckman Coulter, Brea, CA, USA) beads in a 1:1 ratio. The ligation product was amplified using 5′-CGCTCAGCGGCCGCAGCATCACCGCCATCAGT-3′ and 5'-

CGCTCAGCGGCCGCGTCGTAGTGCGCCATCAGT-3′ primers for 12 cycles and was cleaned with AmpureXP beads. A T7 promoter was added by performing a PCR reaction with primer 5'-

GGATTCTAATACGACTCACTATACGCTCAGCGGCCGCAGCATCACCGCCATCAGT-3′ for 12 cycles and was cleaned with AmpureXP beads. Both PCR reactions were set up using 2X KAPA HiFi HotStart ReadyMix (2X) (Roche Diagnostics, Indianapolis, IN, USA) with conditions: 98°C for 2.5 min, 98°C for 20 sec, 55°C for 30 sec, and 72°C for 30 sec. About 500 ng of cleaned PCR product was used in an *in vitro* transcription reaction using a MEGAshortscript kit (Ambion, Carlsbad, CA, USA) (7). Hybrid capture baits were purified using Monarch RNA cleanup kit (New England Biolabs, Ipswich, MA, USA), quantified and stored at −80°C in small aliquots. NGS library preparation of the clinical specimens was performed by Nextera Flex (Illumina Inc., San Diego, CA, USA) or NEBNext UltraII FS DNA Library Prep Kit for Illumina with some modifications. For the Flex kit, the enrichment PCR was performed with conditions: 68°C for 3 min, 98°C for 3 min for one cycle, and 98°C for 45 s, 62°C for 30 s, 68°C for 2 min for 15 cycles, and a final extension for 68°C for 1 min. For the UltaII kit, the fragmentation reaction was set up with up to 26-µL volume of available sample DNA on a thermocycler with conditions: 37°C for 5 min, 65°C for 30 min. The library enrichment PCR was set up with Q5 polymerase with conditions: 98°C for 30 s, 98°C for 10 s, 65°C for 75 s for 20 cycles to obtain sufficient material for baiting procedure.

### Hybridization capture

The NGS library generated from primary specimen was hybridized to capture baits as described in Singh et al. (7) with the following modifications. The samples were denatured along with blocking oligos (both DS adaptor and T7 oligonucleotides), followed by overnight hybridization. The hybridized samples were eluted and amplified for 12 cycles using KAPA Library Amp Primer Mix (Roche Diagnostics, Indianapolis, IN, USA) and were cleaned by AmpureXP beads using 1:0.8 (sample:beads). Last, the samples were subjected to a second hybridization reaction to improve enrichment efficiency. The complete flowchart of the protocol is outlined in Fig. 1. The specimens selected for hybridization capture were based on specimen availability and Ct values.

### Bioinformatics

The baited libraries pre- and post-enrichment were sequenced using Illumina MiSeq 2 × 250-cycle paired-end sequencing. Reads were pre-processed by removing potential adapters using BBDuk from the BBMap from the Joint Genome Institute. The reads were classified by Kraken2 version 2.1.2 (15). The prebuilt database minikraken2_v2_8GB_201904 was used as reference for classification. Kraken2 output was used as the input in Krona tools for plotting the classification (16). Legionellales order-specific read-pairs were extracted for downstream analyses using the extract_kraken_reads (available at https://github.com/jenniferlu717/KrakenTools/) (17). The percent enriched reads were dependent on the reference genome. The percentage is derived from the number of reads identified as *L. pneumophila* vs total number of reads in a data set using Kraken2. As for the different reference genomes used for this analysis, *Legionella* species can be highly heterogenous across different strains, and to maximize the number of genomic sites assessed for single-nucleotide polymorphism (SNP) counts, it is always better to use closely related and high-quality genomes as references. Bwa mem v0.7.17-r1188 (18) was used to map the Legionellales-specific reads over closely related reference *L. pneumophila* strain genomes retrieved from public databases or sequenced and assembled internally from our collection. The reference genomes 2018-clin-CHO, from a related clinical case isolated in 2018 (Table 1; Fig. 4), and *L. pneumophila* Toronto 2005, a high-quality closed genome (Table 2; Fig. 5), were used in the analysis. We selected these references by using Mash searches between the raw reads and a collection of reference genomes downloaded from NCBI. 2018-clin-CHO was *de novo* assembled from raw reads using Unicycler v 0.4.9b with polishing (19). Samtools v1.15.1 was used to sort the reads, remove duplicates, and extract the depth of coverage metrics (20). An in-house script was used to generate genomic consensus sequences for SNP matrix comparisons between samples. A valid position was defined as having a minimum of 10X for depth of coverage, mapping and basecall quality of phred 20 or above, and 95% allele frequency agreement for the state of every nucleotide in the consensus genome. Any position not reaching these requirements was assigned as unknown state (N) and not counted as differences for the SNP counts.

## RESULTS

### *Legionella* baits provide enrichment in spiked sputum specimens

**Fig 2:**
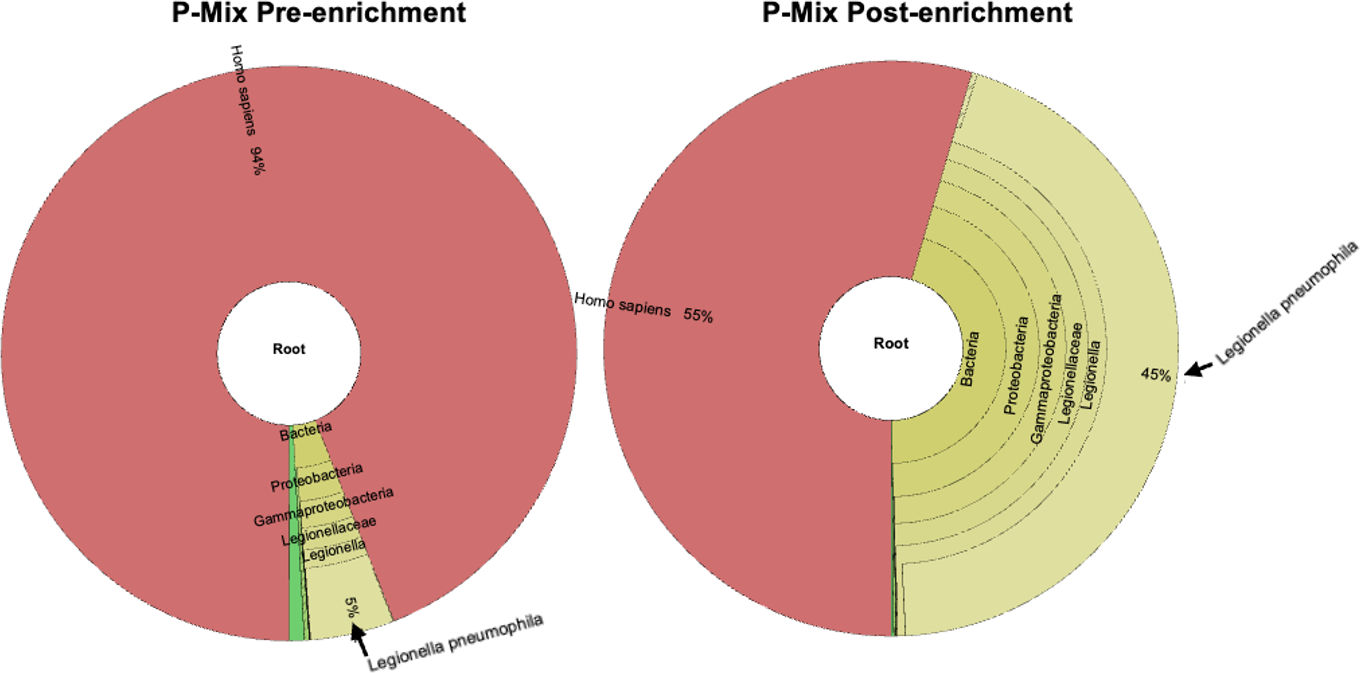
Taxonomic classification of the spiked sputum Pmix pre- and post-enrichment. The arrows indicate *L. pneumophila* reads classified using Kraken2 metagenomics. The resulting proportion of classified reads was plotted by Krona tools.

In a pilot study to evaluate enrichment of *Legionella* in spiked sputum specimens, an NGS library was generated from two mock samples Pmix and Kmix, where purified DNA from *Legionella*-negative sputum was spiked to a final concentration of 5% with genomic DNA from *L. pneumophila* Philadelphia (ATCC 33152) and *L. pneumophila* Knoxville (ATCC 33153), respectively. The hybrid capture protocol was based on our previous method used for STEC enrichment from stool specimens (7) using a two-strain bait. The relative proportion of *L. pneumophila* reads improved from 5% to 45% after enrichment by RNA baits in the Pmix sample (Fig. 2, black arrows). Similarly, the abundance of *L. pneumophila* reads increased from 5% to 42% in the Kmix sample (data not shown). These results provide the proof of concept and suggest that this assay could be used to improve the abundance of *L. pneumophila* DNA in clinical specimens available for WGS analysis.

### Improved hybrid capture by RNA baiting for *L. pneumophila*

**Fig 3:**
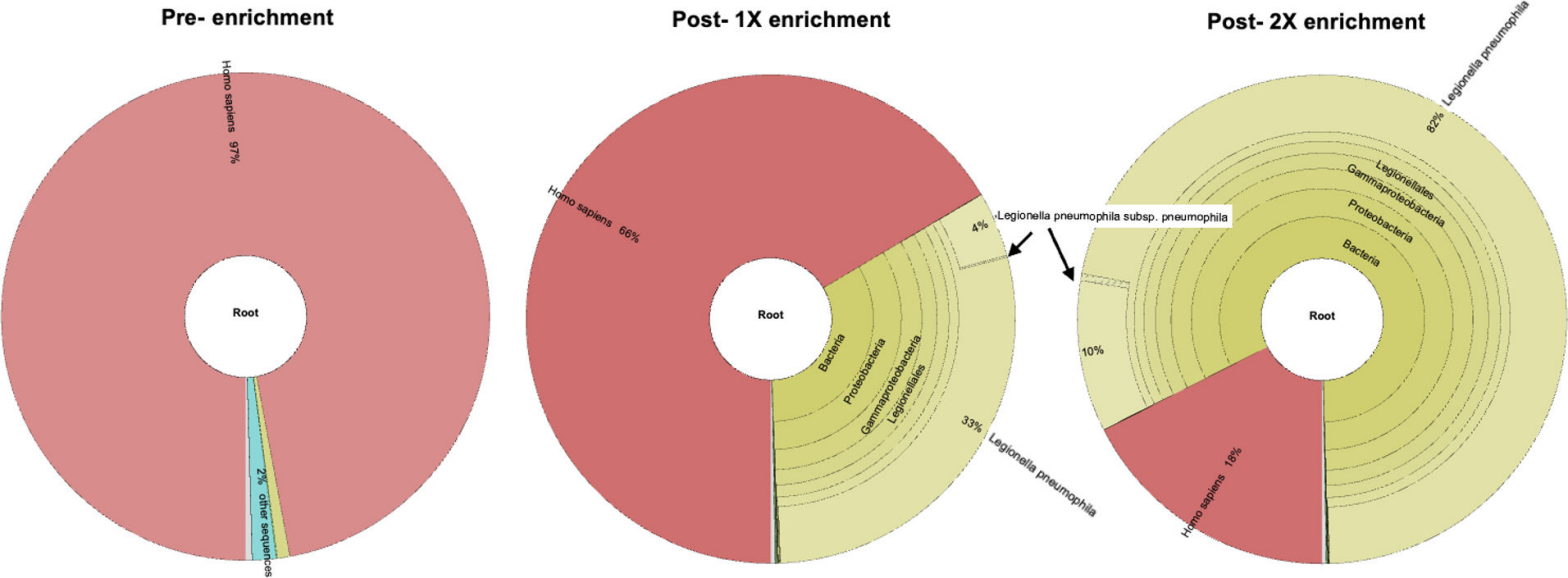
Double enrichment (2X) significantly improved the number of *Legionella* reads in clinical samples as compared to single enrichment (1X) as shown in specimen 1 from Table 1 (black arrows). Reads were classified using Kraken2 and were plotted using Krona tools.

To capture diverse clades of LPSG1, a five-strain bait was generated by mixing equal proportions of five genomes (see Materials and Methods). We tested this hybrid capture protocol in LPSG1-positive clinical specimens. Although single step-enrichment (1X) resulted in significantly higher number of *Legionella* reads as compared to the unenriched control (specimen 1 is shown pre-enrichment: <1%; post-1X enrichment: 33%; Fig. 3, black arrows), there remained a large contamination of human reads. As a result, we did not obtain sufficient *Legionella* read depth post-enrichment for any meaningful bioinformatic analysis.

**TABLE 1:** Relative enrichment of sputum specimens compared to the unenriched controls^*c*^

Specimen	Ct^***b***^ (LPSG1)	Pre-enrichment % *Legionella* reads	Post-enrichment % *Legionella* reads	Fold enrichment	Avg. depth (median)	Genome coverage (%)
1	23	0.05100	81.98	1,607	217 X	97.58
2	20	0.61000	66.01	108	480 X	96.37
3	24	0.16000	84.32	527	333 X	97.18
4	23	0.62000	74.35	120	672 X	91.02
5	26	0.01500	85.57	5,705	71 X	91.02
6	23	0.19000	18.74	99	83 X	89.09
7	25	0.06900	53.91	781	115 X	87.12
8	26	0.09700	27.63	285	60 X	90.93
9	30	0.09100	65.91	724	62 X	91.02
10	31	0.13000	21.63	166	23 X	88.87
11	25	0.14000	20.37	146	13 X	80.94
12^*a*^	32	0.00023	17.33	75,370	11 X	91.43
13	28	0.04100	78.52	1,915	7 X	87.54
14	30	0.02100	38.11	1,815	7 X	93.30
15	25	0.23000	17.50	76	5.2 X	73.60
16	29	0.00027	43.15	159,812	3 X	60.53
17	30	0.12000	31.00	258	1.8 X	44.12
18	34	0.06200	5.04	81	2 X	60.60
19	29	0.02100	56.95	2,712	1 X	53.56
20	32	0.11000	4.21	38	1 X	28.50
21	29	0.00360	11.68	3,244	0.5 X	25.44
22	29	0.04000	15.14	379	1 X	44.39

^*a*^ Sample without a matched pure isolate.

^*b*^ Ct = LPSG1 real-time PCR cycle of threshold values.

^*c*^ Retrospective testing of specimens selected from multiple outbreaks since 2016 and mapped to the *L. pneumophila* 2018-clin-CHO to generate depth and genome coverage.

We next tested the same specimens with two consecutive rounds of hybridization and enrichment, which resulted in a significantly higher number of *Legionella* reads (specimen 1, post-1X enrichment: 33%, post-2X enrichment: 82%; Fig. 3, black arrows) and lower number of human reads. The hybridization steps were improved by adding blocking oligos (DS adaptor and T7 oligos) to the reaction prior to denaturation, which also helped improve the RNA-DNA hybridization process. A ligation-based NGS library preparation using the UltraII kit from NEB further improved the yield of *Legionella* reads in a sequencing run as compared to the transposon-based Illumina Flex kit (data not shown). This improved workflow for *Legionella* hybrid capture prior to direct WGS is outlined in Fig. 1.

### Significant enrichment of *Legionella* reads in sputum specimens observed using improved hybrid capture

The 30 LPSG1 real-time PCR-positive sputum specimens with Ct values ranging from 20 to 38 (Tables 1 and 2, column 3) were tested with this approach. A matched pure isolate was recovered from 27 of these specimens for which WGS was performed. The sputum specimens were subjected to double enrichment by baiting using a five-strain bait and the additional modifications described above. Each sample was sequenced prior to hybrid capture as a pre-enrichment control. The proportion of reads classified as *Legionella* improved considerably after enrichment, and this increase ranged from 38- to 209,000-fold (Tables 1 and 2, column 6). The specimens with lower Ct values generally showed better average depth and genome coverage after read mapping to a reference genome. In some exceptional cases, the DNA quality of the primary specimen may have adversely affected the enrichment. For example, both specimen 7 and specimen 11 in Table 1 have a Ct value of 25, but specimen 7 resulted in 115X average depth and 87.12% genome coverage after enrichment, and specimen 11 resulted in only 13 X average depth and 80.94% genome coverage after enrichment. The resulting insufficient number of Legionella reads impacts the downstream bioinformatic analysis, which can ultimately fail to resolve the outbreak. The specimens that indicated ~40X average depth and ~80% genome coverage resulted in sufficient valid positions for downstream WGS bioinformatic analysis (Tables 1 and 2, column 7 and column 8).

**TABLE 2:** Relative enrichment of sputum specimens compared to the unenriched controls^*c*^

Specimen	Ct^*b*^ (LPSG1)	Pre-enrichment % *Legionella* reads	Post-enrichment % *Legionella* reads	Fold enrichment	Avg. depth (median)	Genome coverage (%)
23^*a*^	26	0.00440	72.08	16,382	111 X	95.78
24	29	0.00061	65.24	106,949	51 X	95.22
25^*a*^	30	0.00039	70.12	179,798	48 X	94.53
26	28	0.00130	65.36	50,279	43 X	91.38
27	38	0.00005	0.80	17,497	0 X	8.49
28	35	0.00004	7.74	209,120	2 X	50.59
29	36	0.00033	0.12	349	0 X	2.64
30	38	0.00017	0.55	3,210	0 X	10.31

^*a*^ Sample without a matched pure isolate.

^*b*^ Ct = LPSG1 real-time PCR cycle of threshold values.

^*c*^ The retrospective samples were selected from multiple outbreaks since 2016 and mapped to the *L. pneumophila* Toronto 2005 to generate depth and genome coverage.

### *Legionella*-positive clinical specimens matched closely to their paired pure isolates

**Fig 4:**
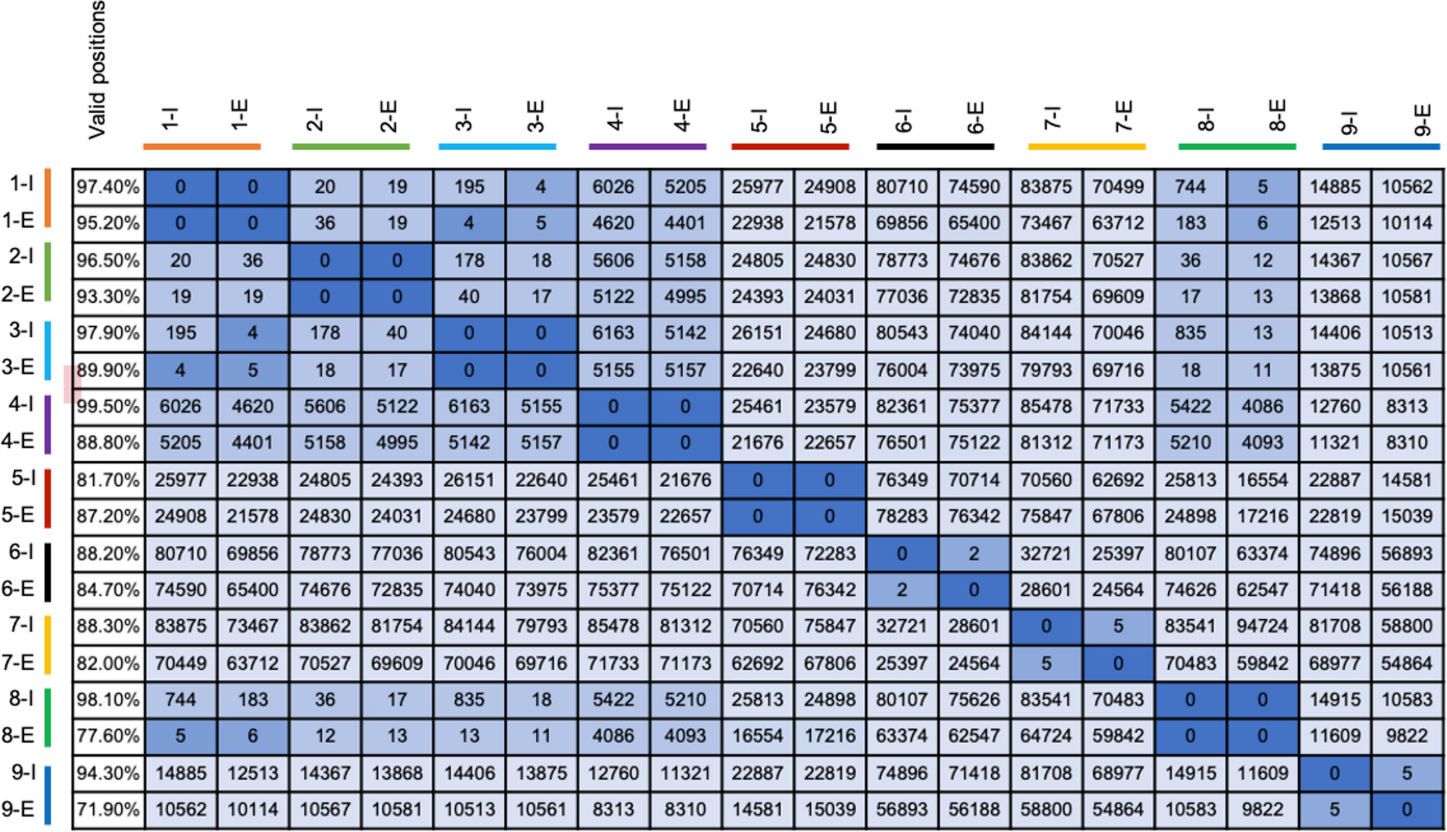
Percent valid positions of genome coverage and SNP matrix of clinical sputum isolates from Table 1. -E, enriched primary specimen; -I, matched pure isolate. *L. pneumophila* 2018-clin-CHO was used as a reference genome. The color bar on the legend indicates the matched enriched (E) and isolate (I) pair.

**Fig 5:**
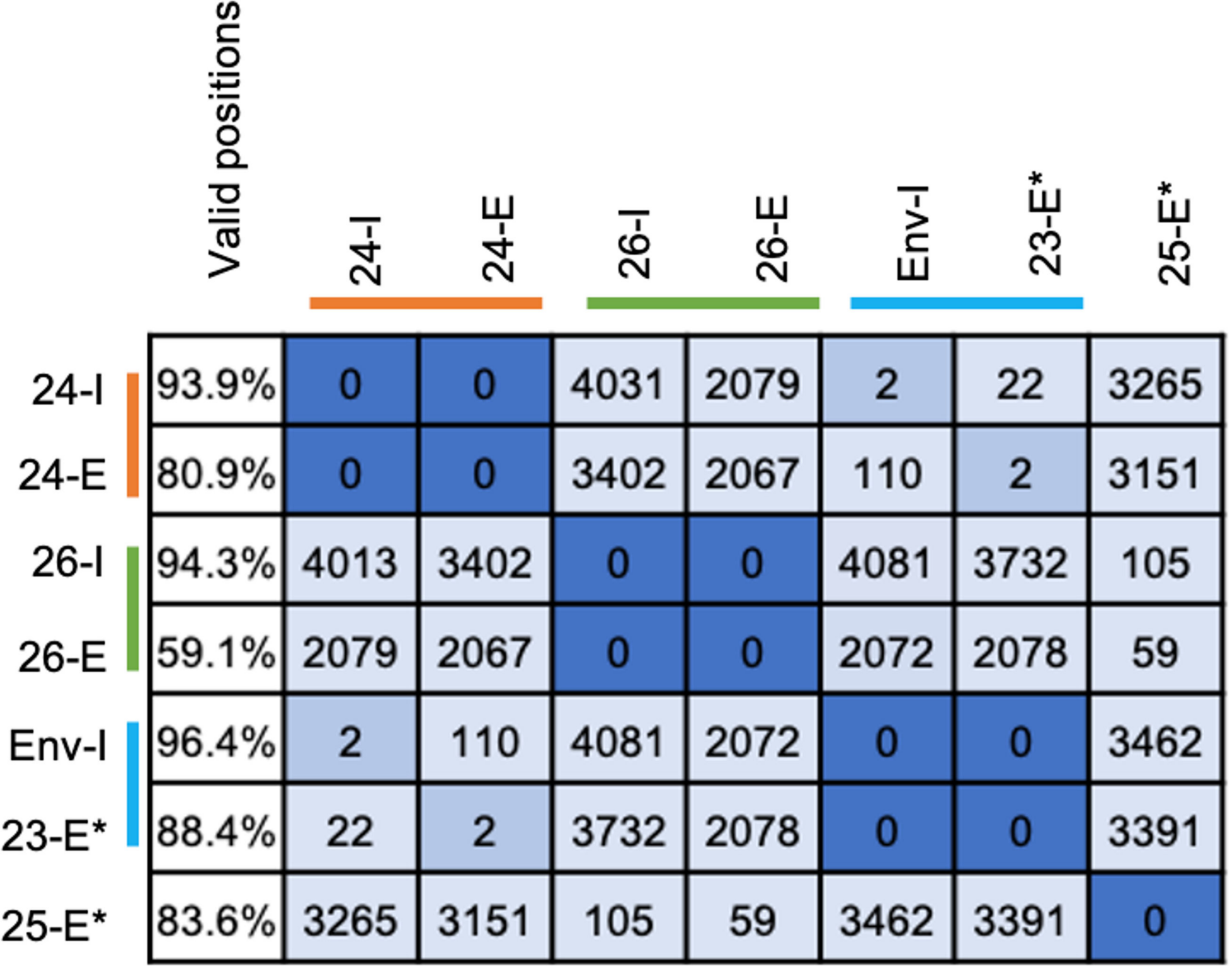
Percent valid positions of genome coverage and SNP matrix of clinical sputum isolates from Table 2 matched to their pure isolates. -E indicates enriched primary sample, -I indicates their matched pure isolate WGS. *L. pneumophila* Toronto 2005 was used as a reference genome. The color bar on the legend indicates the enriched (E) and isolate (I) pair. * = sample without a pure isolate.

Clinical sputum specimens for which remaining specimen was available of variable Ct values were processed using hybrid capture as described above and were compared with their corresponding WGS data from cultured isolates. Bioinformatic analysis resulted in the SNP matrix, which was generated from this specimen comparison using *L. pneumophila* 2018-clin-CHO as a reference genome (Table 1; Fig. 4) and *L. pneumophila* Toronto 2005 as a reference genome (Table 2; Fig. 5). Valid genomic positions were calculated as genome positions with 10X of above of depth of coverage and 95% allele agreements after quality filtering. A SNP matrix was generated from all enriched specimens (E) along with the pure isolate (I). Of the 30 sputum specimens, the SNP matrix from 13 enriched specimens (E) with sufficient valid genomic positions along with their pure isolate (I) is shown in Fig. 4 (specimens 1–9) and Fig. 5 (specimens 23–26).

Data from enriched specimens (E) and their pure isolate (I) are shown as pairs next to each other, connected by a color bar. When compared, all enriched specimens differed from their respective pure culture counterpart by only zero to five SNP (Fig. 4 and 5, dark blue), indicating that this enrichment method can be used to confirm or refute potential linkages between cases during the course of an investigation. It should also be noted that this analysis depends on SNP counts for the parts of the genome that are present. The higher the percent of valid genome positions, the more confidence there is in the accuracy of the final SNP counts. Sometimes, we observed SNP differences between closely clustered genomes, e.g., in two instances of five SNP differences (Fig. 4, samples 7 and 9) and in one instance of two SNP differences (Fig. 4, sample 6) between pairs of enriched and isolate WGS that would be expected to have been identical. Upon manual inspection, we found that most differences were closely clustered together and could have been caused by PCR artifacts during the amplification steps. Nevertheless, we only encountered this issue in three of the sample comparisons performed, and the SNP counts were still very low and in the typical range considered to be related by most analyses, warranting a closer look for possible links during an outbreak investigation.

### Hybrid capture provided additional information on culture-negative sputum specimens

We next tested three sputum specimens where we were unable to recover a bacterial culture isolate and sufficient specimen was available with the lowest Ct values (Table 1, sample 12^a^, and Table 2, sample 23^a^, sample 25^a^). Specimen 12^a^ (Ct 32) resulted in 75,370-fold enrichment but resulted in insufficient valid positions for any downstream analysis (Table 1). Specimen 23^a^ (Ct 26), which was collected in the year 2016, resulted in a 16,382-fold enrichment and 88.4% valid positions (Table 2; Fig. 5). It was compared to all the outbreak isolates from 2016 in our database and a perfect match was identified to Env-1 with a 0 SNP difference (Table 2; Fig. 5), indicating a potential source of infection. Specimen 25^a^ (Ct 30) was collected in the year 2018 and resulted in 179,798-fold enrichment but did not match to any isolate in our database despite 83.6% valid positions, indicating it was not linked to any ongoing or past outbreak at the time of collection (Table 2; Fig. 5).

### Real-time PCR testing for South Bronx 2015 autopsy specimens

Thirteen suspect cases during the SB outbreak for which there were autopsy specimens were examined by real-time PCR. LPSG1 DNA was detected in 19 fresh lung tissue specimens, 16 formalin-fixed lung tissue specimens, 1 urine specimen, and 3 vitreous fluid specimens (Table 3, patient A–J). One formalin-fixed tissue was inconclusive for LPSG1 DNA due to levels of DNA at or near the limit of detection (LOD) of the assay. One formalin-fixed tissue was determined to be positive for *L. pneumophila* DNA but negative for LPSG1 potentially due to the LOD of LPSG1 target. A comparison of real-time PCR Ct values between the paired fresh and formalin-fixed tissues from the same patient revealed an average >1 log decrease in Ct value (increased bacterial load) for fresh tissues.

**TABLE 3:** Comparison of LPSG1 real-time PCR results (Ct values) of autopsy specimens collected from patients (A–J) during the SB 2015 outbreak^*c*^^,^*^d^*

	Fresh lung		Formalin-fixed lung			
Patient	Right (Ct)	Left (Ct)	Right (Ct)	Left (Ct)	Urine (Ct)	Vitreous fluid (Ct)
A	35^*a*^	35^*a*^	36	36		-
B	33	24^*a*^	34	28		
C	25^*a*^**^,*b*^**	29^*a*^	32	35		36
D	24^*b*^	17	22	31		
E	33^*b*^	26^*b*^	37	Inc.		-
F	21^*b*^	22^*b*^	28	27	38	35
G	34	22	38	37	-	-
H	26	30	38	35	-	-
I	-	35	-	37	-	38
J	32	32				

^*a*^ Culture-positive and WGS data available.

^*b*^ Samples used for hybrid capture.

^*c*^ Ct = cycle threshold; Inc. = inconclusive; (-) = negative.

^*d*^ Three additional patients for which lung tissue and vitreous fluid were received were negative for all testing and were not shown (patients K–M).

### Culture of fresh lung autopsy tissue for *L. pneumophila* serogroup 1

Pure *L. pneumophila* isolates were recovered from fresh lung autopsy specimens from 3 (patients A, B, C) out of 10 PCR-positive patients (patients A–J, five total specimens), which are highlighted as ^a^ in Table 3. These specimens from three cases were subjected to WGS and were identified as a part of the SB outbreak. Overall, the utilization of acid washing yielded the greatest recovery of *Legionella* from culture for these specimens. Heat treatment and ethanol treatments were also utilized to overcome swarming organisms present, but the treatments did not provide the ability to culture some specimens. Utilization of both BCYEα and BCYEα+ PAV media proved to be the most successful for *L. pneumophila* isolation in these specimens. Additional attempts utilizing enrichment and dilution plating were performed without success to isolate *L. pneumophila* from the remaining specimens (data not shown). Isolates could not be recovered from the remaining seven LPSG1 PCR-positive autopsy patients, and a definitive WGS link to the SB outbreak could not be made. Attempts to conduct WGS directly from these autopsy specimens without culture resulted in insufficient recovery of *Legionella*-specific reads due to the high prevalence of the host genome.

### Genome level resolution on South Bronx autopsy specimens using hybrid capture

**Fig 6:**
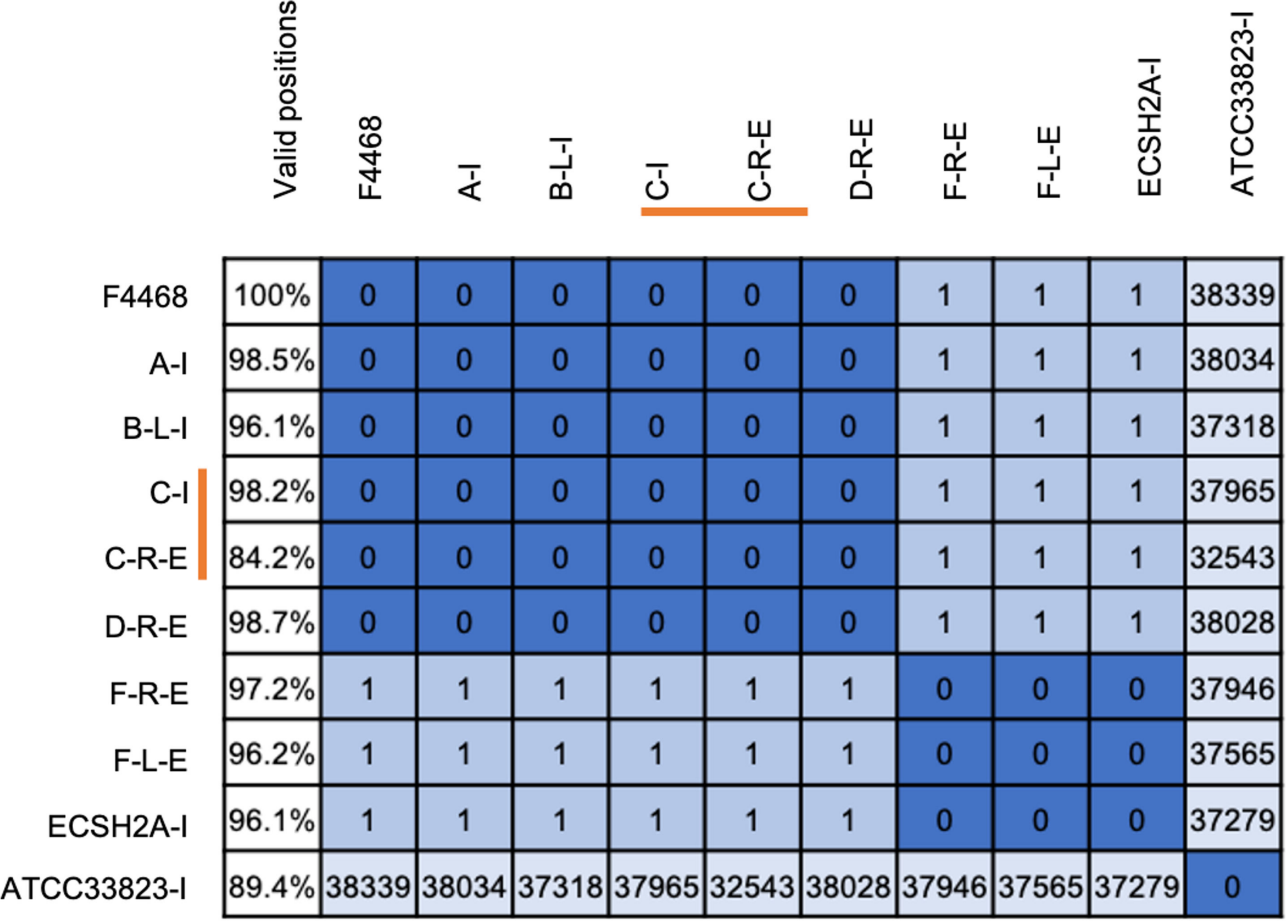
Percent valid positions and SNP matrix of South Bronx autopsy specimens from Table 3 compared to the outbreak strain F4468. The legends indicate the patient numbers A–F, enriched (-E), pure isolate (-I), left lung (-L), and right lung (-R). The color bar indicates an isolate (I) and enriched sample (E) pair.

We tested six autopsy specimens from four cases (patients C, D, E, F, Table 3; Fig. 6) that were still available in our 2015 SB outbreak specimen collection to identify a direct link to the source. Four specimens were successfully enriched for *L. pneumophila* with at least 84% of the genome positions passing threshold for SNP analyses (column 1, Fig. 6). Patient D right lung (D-R-E, row 6, Fig. 6) and patient C right lung (C-R-E, row 5, Fig. 6) had 0 SNP difference with the culture isolate of patient C (C-I, row 4, Fig. 6) and the SB 2015 outbreak strain F4468 (row 1, Fig. 6), indicating a definitive link. Both the right and left lung of patient F (F-R-E and F-L-E, rows 7 and 8, Fig. 6) showed one SNP difference when compared to the outbreak strain F4468 (row 1, Fig. 6). Interestingly, one might conclude that this single SNP in patient F was a spontaneous mutation that occurred in the patient after being infected with the outbreak strain. However, subsequent comparisons with another environmental sample ESCH2A-I (row 9, Fig. 6) taken from a cooling tower during the 2015 outbreak showed a 100% match with the patient strain, proving that this patient was not part of the main outbreak but rather linked to a different cooling tower located 7 miles away from which an environmental source isolate matched (10). We also compared an unrelated strain, ATCC 33823-1 (row 10, Fig. 6), which was found to be thousands of SNPs different.

Both the right lung (Ct 33) and the left lung (Ct 26) of patient E (Table 3) did not result in sufficient enrichment to give an accurate SNP assessment. Poor quality DNA due to improper specimen collection or prior antibiotic treatment of the patient may have attributed to this failure.

## DISCUSSION

In this study, we developed *L. pneumophila* hybrid capture method that utilizes an RNA baiting approach followed by WGS, which robustly enriches the *L. pneumophila* reads directly from clinical specimens. We initially evaluated this approach on a group of sputum specimens with a diverse range of Ct values for LPSG1 by real-time PCR. These specimens revealed enrichment for *Legionella* reads (up to 209,000-fold). In a subset of these with matched isolates from the same initial specimen, the enriched specimen samples were then determined to be zero to five SNP different. The success of enrichment was found to be directly related to the pathogen load in the DNA from primary specimens indicated by Ct values of 30 or below.

This approach was developed to provide a powerful resource especially for unsolved outbreaks where no *Legionella* isolate can be recovered from available specimens. Three sputum specimens (specimen 12^a^ in Table 1, and specimens 23^a^ and 25^a^ in Table 2) from cases with previously unconfirmed matches to outbreaks or sources were also tested. Using this enrichment protocol, one case (specimen 23^a^, Table 2; Fig. 5) was determined to be conclusively linked to an environmental source outbreak isolate; whereas, the second case (specimen 25^a^, Table 2; Fig. 5) did not match to a known outbreak isolate. One case (specimen 12^a^, Table 1) did not have sufficient *Legionella* reads and failed further analysis.

For the SB 2015 outbreak, we had a group of 13 suspect cases for which we initially received a variety of autopsy specimens. We screened all specimens by real-time PCR, which found 10 of the 13 to be LPSG1 PCR-positive. The vitreous fluid and urine were novel specimens and were added as an assessment for future outbreak investigations. Although positive for a few of the cases, the Ct values were high compared to lung tissue samples and were also below the detection limit of the hybridization capture assay described here.

In this study, we tested culture-negative LPSG1 PCR-positive autopsy specimens (patients D and E) and a culture-positive LPSG1 PCR-positive autopsy specimen (patient C) that were all associated with the SB 2015 outbreak. After enrichment, we can now definitively conclude that patient C specimen matched to its pure isolate sample as well as to the SB 2015 outbreak strain. Patient D, with no pure culture isolate, was also determined to be definitively linked to the cooling tower source that caused the SB 2015 outbreak (Fig. 6). In contrast, two additional autopsy specimens from one case (patient F, Fig. 6), earlier thought to be part of the SB outbreak, were determined to be linked to a different cooling tower (ESCH2A) located 7 miles away (10).

New NGS approaches for nonculturable, low pathogen load, or difficult to culture pathogens are being developed and assessed. An example is our earlier work focused on the challenging-to-identify, non-O157 Shiga toxin-producing *E. coli* from stool species (7). Additionally, a selective whole genome amplification (SWGA) for *Treponema pallidum* has shown robust direct WGS of specimens containing very low pathogen load, which has been challenging until now (21). In a similar study focused on cutaneous leishmaniasis, for which there is a technical challenge of isolating and culturing parasites from patient lesions, SWGA provided a relatively simple method to generate *Leishmania* genomes directly from patient specimens, unlocking the potential to link parasite genetics with host clinical phenotypes (22). In another study, a new strategy to identify mixed infections and minority variants in *Mycobacterium tuberculosis* by WGS is described. The objective was the direct detection in patient sputum so that minority populations of resistant strains could be identified, and the most appropriate treatment could be utilized as soon as possible. This approach used a platform for capturing *M. tuberculosis*-specific DNA that was designed to enrich the clinical specimen and obtain quality sequences (23). It is expected that continued progress for direct specimen WGS will continue to be refined and will play an important role in clinical diagnosis and public health in the near future.

Overall, the approach described here has shown success for about half of primary specimens tested including sputum and fresh lung tissues collected as part of autopsies. The pathogen load in the initial specimen collected is an important factor for the success of this testing. In the future, long-read sequencing approaches instead of short-read sequencing for hybrid capture will be tested, which may help improve the genome coverage. The development of new techniques and strategies like hybrid capture for the clinical diagnosis of *Legionella* can provide a significant improvement for the surveillance and management of LD. In recent years, there are growing health risks associated with LD due to global challenges like urbanization, climatic changes, and economic developments. New approaches like the one presented in this study offer an additional tool for LD outbreak investigations and may help solve previously unanswered questions from past investigations.

## Data Availability

Raw Illumina reads for enriched samples and their controls were submitted to the SRA database at NCBI under BioProject accession number PRJNA1066847. The % pre-enrichment and % post-enrichment columns in Tables 1 and 2 were derived from a run where both the control and enriched samples were processed together. However, some enriched samples were sequenced more than once, and data were pooled to improve depth of sequencing in Fig. 4 and 5. The SRA submission included the enriched pooled data sets. The Raw Illumina reads from autopsy isolates were downloaded from PRJNA345011 (New York City outbreak), PRJNA1066847, and PRJNA483184 (Health Facility study). Genomes for the *Legionella pneumophila* reference strains Knoxville (CP021266.1), Toronto 2005 (CP012019.1), Paris (CR628336.1), Philadelphia-1 (CP013742.1), F4468 (CP014759.1), and Dallas (CP017458.1) were downloaded from GenBank. The LegioCluster software (version1.0.0) is available at GitHub (https://github.com/WHaasNY/LegioCluster).
